# OnabotulinumtoxinA in chronic migraine: is the response dose dependent?

**DOI:** 10.1186/s12883-022-02742-x

**Published:** 2022-06-13

**Authors:** Ali Zandieh, Fred Michael Cutrer

**Affiliations:** 1 Department of Neurology, University of Wisconsin, 1685 Highland Avenue, Madison, WI 53705 USA; 2 Department of Neurology, Mayo Clinic, 200 First Street SW, Rochester, MN 55905 USA

**Keywords:** Headache, Chronic migraine, OnabotulinumtoxinA, Migraine treatment, Dose response

## Abstract

**Background:**

OnabotulinumtoxinA has been widely used for control of chronic migraine. The aim of the current study was to evaluate the efficacy of different doses of the onabotulinumtoxinA therapy in patients with chronic migraine.

**Methods:**

This is a retrospective paired comparison study on patients with chronic migraine who received at least 3 rounds of 150 units of onabotulinumtoxinA followed by at least 3 rounds of 200 units of onabotulinumtoxinA. The data from the patient-reported questionnaires about headache days, severe headache days and wearing off periods were reviewed.

**Results:**

A total of 175 patients were included in this study. The headache days and severe headache days decreased from 13.62 ± 10.79 and 5.88 ± 6.73 to 11.02 ± 10.61and 4.01 ± 4.89 days, after increase in the onabotulinumtoxinA dose, respectively (*P* < 0.001 for both comparisons). The favorable effect of the 200 units compared to the 150 units of the onabotulinumtoxinA, was independent from the headache location and the duration of the onabotulinumtoxinA 150 units therapy; and persisted as patients continued to receive the higher dose of onabotulinumtoxinA. Increase in the onabotulinumtoxinA dose was also associated with a decreased wearing off period (*P* < 0.05).

**Conclusion:**

We found that increase in the onabotulinumtoxinA is associated with fewer headache and severe headache days. Future randomized clinical trials are needed to confirm the dose-dependent response to onabotulinumtoxinA.

## Background

Headache is among the most common chief complaints in medicine and neurology [1]. Globally, headache is the second leading cause of years living with disability [2]. Migraine as the second most common primary headache disorder, accounts for the majority of headache related disability and clinic visits [3]. It affects around 10 % of the population and imposes a huge burden on society [2, 3].

Epidemiologic studies have shown that around 2 % of the adult population suffers from chronic migraine, which is distinguished from episodic migraine by the number of the headache days [4, 5]. Chronic migraine is far more debilitating and is associated with higher rates of anxiety and depression [6]. Patients with chronic migraine are less successful in their career and have lower income [6]. The higher burden of chronic migraine further necessitates the need for better treatment in this subgroup of patients. Unfortunately, the frequent use of acute pain medications like non-steroidals, triptans, or opioids, may complicate the treatment in patients with chronic migraine [7, 8].

OnabotulinumtoxinA has been found to be effective against a variety of painful disorders, including chronic migraine, myofascial and neuropathic pain [9–11]. In 2010, United States food and drug administration approved onabotulinumtoxinA as a preventative therapy for chronic migraine (https://www.accessdata.fda.gov/drugsatfda_docs/label/2011/103000s5236lbl.pdf). It is associated with improvement in several headache related measures, like frequency of headache and migraine days, pain intensity, headache related disability and quality of life [11–13]; and its beneficial trend persists over one year after the initiation of the therapy [14, 15]. Earlier therapy with onabotulinumtoxinA is associated with more favorable and sustained response [16]. Although the role of onabotulinumtoxinA in treatment of chronic migraine is well studied, it is not clear if its beneficial effect is dose dependent. In the Phase 3 REsearch Evaluating Migraine Prophylaxis Therapy (PREEMPT) trial, providers were allowed to increase the administered dose of onabotulinumtoxinA by 40 units which roughly equals 25% of the minimum injected dose [11]. Similarly, headache providers commonly modify the onabotulinumtoxinA dose, even though, thus far, there is no compelling evidence of a dose dependent response [17].

The highly disabling nature of the chronic migraine dictates the need for more effective treatments. Overall, the prophylactic migraine treatments are underutilized, although their use results in lower healthcare costs [18, 19]. OnabotulinumtoxinA is not an exception here, and patients may benefit from higher doses of onabotulinumtoxinA. The aim of the current study is to better characterize the role of the onabotulinumtoxinA therapy in chronic migraine and determine whether its effect is dose dependent.

## Methods

### Study population

This is a retrospective paired comparison study conducted on patients who were receiving onabotulinumtoxinA for chronic migraine at Mayo Clinic, Rochester, MN.

Chronic migraine was defined as ≥15 headache days per month, with headaches having migrainous features on ≥8 days per month, averaged over the previous three months (https://ichd-3.org/1-migraine/1-3-chronic-migraine/). Charts of the patients who visited our onabotulinumtoxinA clinic from 10/1/2020 till 12/31/2020 were reviewed. In order to be eligible for this study, patients should have at least three rounds of onabotulinumtoxinA 150 units followed by at least three rounds of onabotulinumtoxinA 200 units. In accordance with the State of Minnesota law, the individuals who declined the use of their medical records for research, were not included. Additionally, individuals with missing data, and those who did not show up within one week before or after the due date for their next onabotulinumtoxinA appointment, were excluded. This study was approved by Mayo Clinic Institutional Review Board (registration number 20–012142).

### OnabotulinumtoxinA injection protocol

**Fig. 1:**
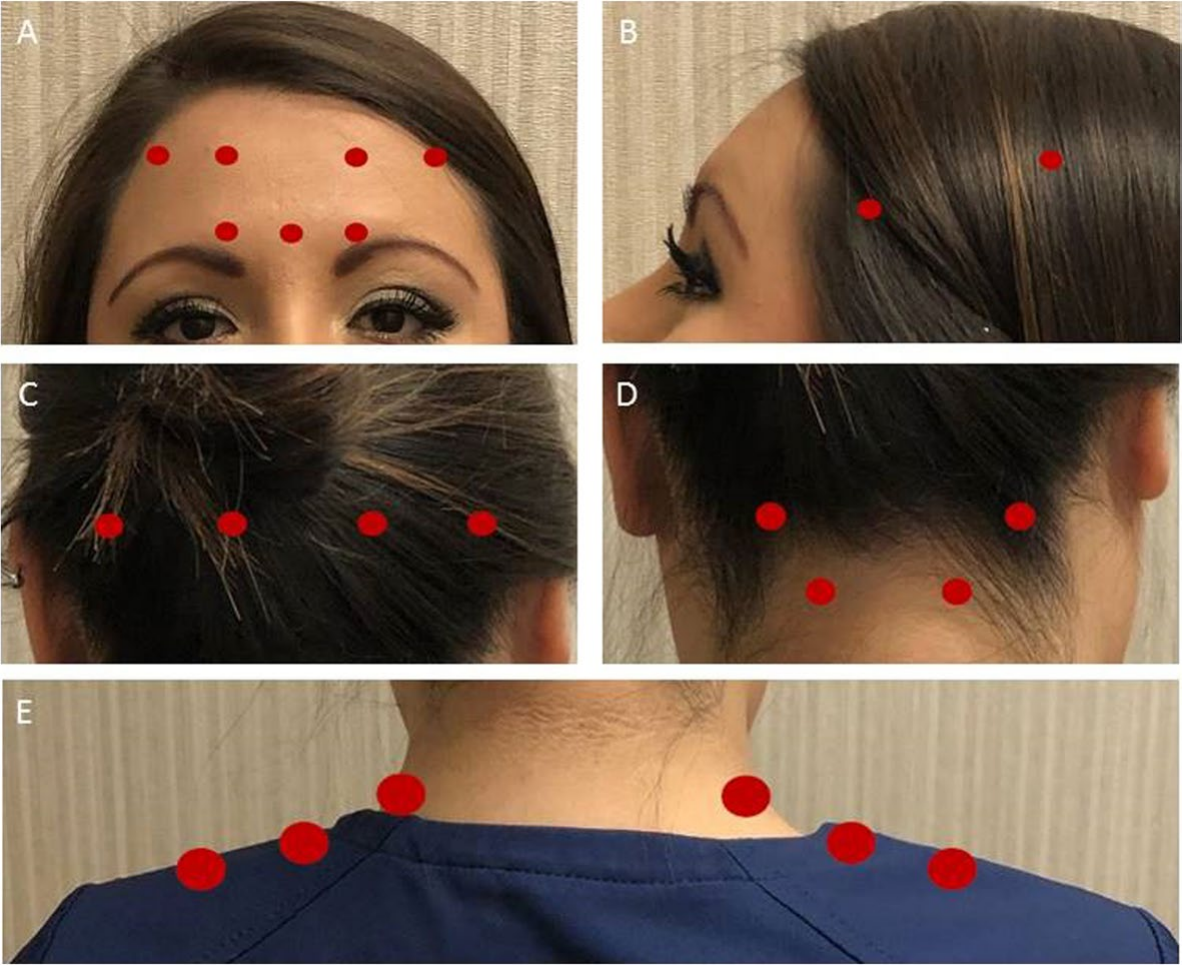
OnabotulinumtoxinA 150 units protocol. **A** 5 units (1 injection site) in procerus muscle, 5 units (1 injection site) in each corrugator muscle, and 5 units (2 injections sites) each frontalis muscle. **B** 12.5 units (2 injection sites) each temporalis muscle. **C** 12.5 units (2 injection sites) each occipitalis muscle. **D** 12.5 (2 injection sites) each splenius muscle. **E** 25 units (3 injection sites) each trapezius muscle

**Fig. 2:**
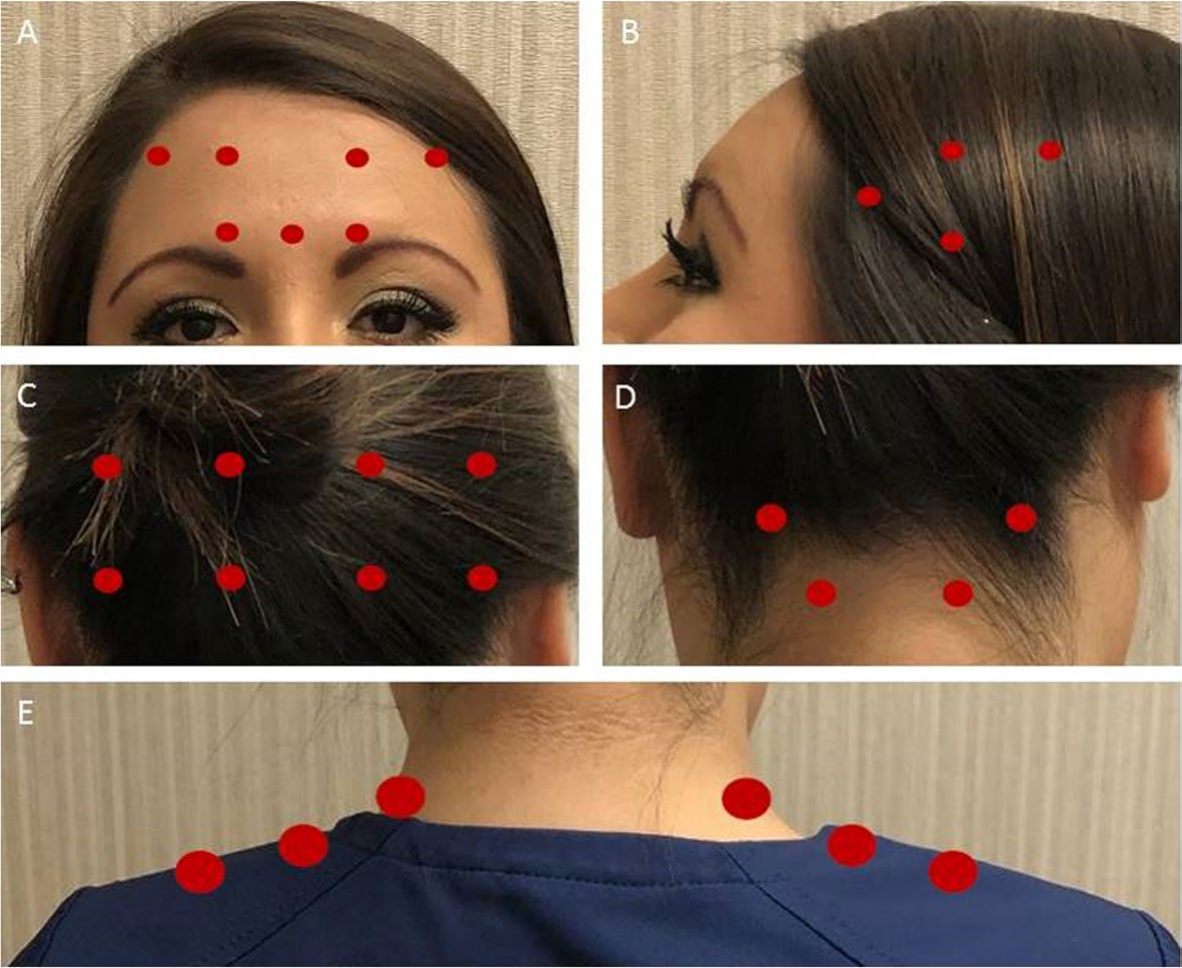
OnabotulinumtoxinA 200 units protocol. **A** 5 units (1 injection site) in procerus muscle, 5 units (1 injection site) in each corrugator muscle, and 5 units (2 injections sites) each frontalis muscle. **B** 25 units (4 injection sites) each temporalis muscle. **C** 25 units (4 injection sites) each occipitalis muscle. **D** 12.5 (2 injection sites) each splenius muscle. **E** 25 units (3 injection sites) each trapezius muscle

At Mayo Clinic in Rochester, for migraine prophylaxis patients start with 150 units of onabotulinumtoxinA. The injections sites and the doses are shown in Fig. 1. In summary, during onabotulinumtoxinA 150 units rounds, patients receive 5 units (1 injection site) in procerus muscle, 5 units (1 injection site) in each corrugator muscle, 5 units (2 injections sites) each frontalis muscle, 12.5 units (2 injection sites) each temporalis muscle, 12.5 units (2 injection sites) each occipitalis muscle, 12.5 (2 injection sites) each splenius muscle and 25 units (3 injection sites) each trapezius muscle. In our clinic, patients have routine follow up appointments nine months after the start of the onabotulinumtoxinA therapy, followed by semiannual clinic visits. During these visits, patients are asked if they are satisfied with their headache control. If not, providers discuss different headache treatment options including an increase in the onabotulinumtoxinA dose. If at least by the third round of the 150 units onabotulinumtoxinA, patients do not reach their self-defined optimal response, the onabotulinumtoxinA dose may be increased to 200 units. The injections sites and the doses for 200 units protocol are shown in Fig. 2. In summary, during onabotulinumtoxinA 200 units rounds, patients receive 5 units (1 injection site) in procerus muscle, 5 units (1 injection site) in each corrugator muscle, 5 units (2 injections sites) each frontalis muscle, 25 units (4 injection sites) each temporalis muscle, 25 units (4 injection sites) each occipitalis muscle, 12.5 (2 injection sites) each splenius muscle and 25 units (3 injection sites) each trapezius muscle. Hence, the extra 50 units are divided and injected evenly into the temporalis and occipitalis muscles.

### Data collection

The Electronic Medical Record system was used to collect the data, which included age, sex, body mass index (BMI), age of onset of migraine, headache location, aura, and family history of headache. The location of the most intense pain was categorized as 1: frontal, 2: temporoparietal, 3: occipitonuchal, and 4: holocephalic. The last clinic note before the start of the onabotulinumtoxinA therapy was used to record the number of the baseline headache days and severe headache days.

The numbers of the different preventative medications that patients were taking at 36 weeks (equal to three rounds) before the increase in the dose and at the time of the increase to 200 units were also collected. The considered preventative medications were propranolol, atenolol, metoprolol, verapamil, topiramate, sodium valproate, gabapentin, amitriptyline, nortriptyline, venlafaxine and calcitonin gene-related peptide monoclonal antibodies.

During the onabotulinumtoxinA injection appointments, patients are required to complete a questionnaire about the total number of the headache days and self-defined severe headache days they have each month. They are also inquired if they feel onabotulinumtoxinA wears off before their next appointment and if yes, how long before the appointment, onabotulinumtoxinA starts to wear off. Patients’ answers to these questions during each round of onabotulinumtoxinA therapy were recorded for the current study.

### Statistical analysis

In order to study the efficacy of the higher dose of onabotulinumtoxinA, paired t-test was used to evaluate the trend of the number of the headache days, severe headache days and length of the wearing off period after the increase in the onabotulinumtoxinA dose. *P* value < 0.05 was considered statistically significant.

## Results

Total of 175 patients were included in this study. The principal characteristics of the individuals are shown in Table 1. The majority of the individuals were female (77.1%). The mean ± standard deviation (SD) age and BMI of the participants were equal to 47.31 ± 13.45 years and 29.72 ± 7.52 kg/m^2^, respectively. Almost one-third of the patients had aura (34.9%) and two-thirds had a family history of migraine (70.9%). The most common headache location was frontal (41.7%), followed by temporoparietal (29.1%), occipitonuchal (16.0%) and holocephalic (13.1%).

**Table 1:** Principal characteristics of the enrolled subjects. (*n* = 175)

Characteristics	Values
Age, year	47.31 ± 13.45
Sex (Female)	135 (77.1%)
BMI, kg/m^2^	29.72 ± 7.52
Age of onset, year	23.15 ± 13.68
Aura	61 (34.9%)
Family history of headache	124 (70.9%)
Baseline headache days	
Total number of headache days	25.85 ± 5.73
Number of severe headache days	15.20 ± 7.96
Headache location	
Frontal	73 (41.7%)
Temporoparietal	51 (29.1%)
Occipitonuchal	28 (16.0%)
Holocephalic	23 (13.1%)

Data presented as mean ± SD, unless otherwise stated

**Fig. 3:**
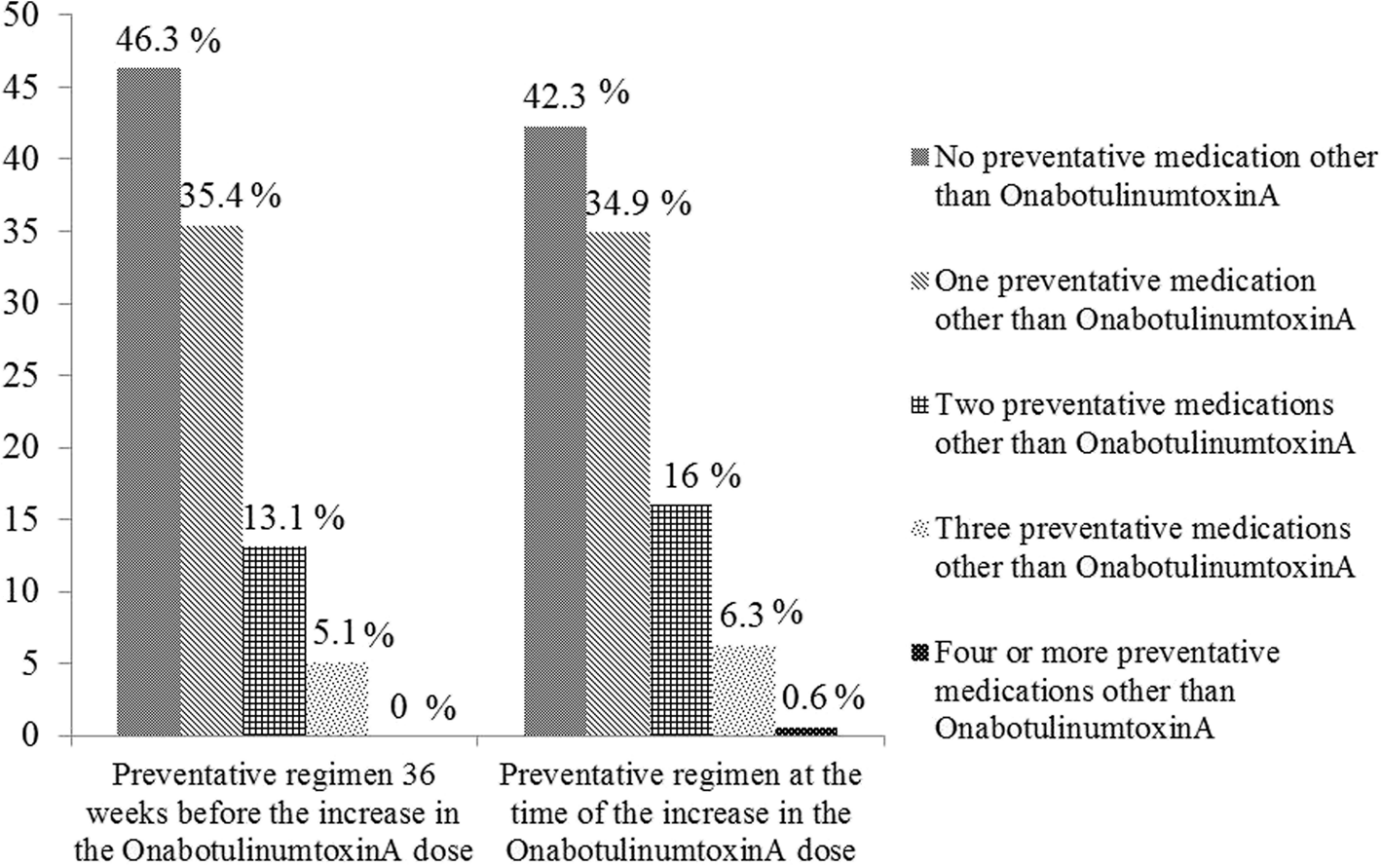
Pattern of the preventative medication use during the course of the study

Figure 3 illustrates the preventative medication use during the course of the study. The percentages of the patients who were not on any preventative therapy other than the onabotulinumtoxinA therapy were not significantly different between 36 weeks before and the time of the increase in the onabotulinumtoxinA dose (46.3%, vs 42.3%, respectively).

Table 2 shows the trend of headache response to onabotulinumtoxinA. Number of the headache days and number of severe headache days significantly decreased after increase in the onabotulinumtoxinA dose. After 3 rounds of onabotulinumtoxinA 200 units, the headache days and severe headache days decreased from 13.62 ± 10.79 and 5.88 ± 6.73 to 11.02 ± 10.61and 4.01 ± 4.89, respectively (*P* < 0.001 for both comparisons). This trend persisted after individuals were stratified based on the duration they had received 150 units of onabotulinumtoxinA (Table 3). Total of 83 patients received at least 8 rounds of onabotulinumtoxinA 150 units (which roughly equals to 2 years). In this subgroup of patients, there was a trend for worsening of the headache for the past several months prior to the increase in the onabotulinumtoxinA dose. This trend not only stopped but also reversed after they were placed on the higher dose of the onabotulinumtoxinA therapy (Table 3). They reported 12.79 ± 9.77 and 6.67 ± 7.48 headache and severe headache days per month, respectively, at the time of increase of the onabotulinumtoxinA dose. These numbers improved to 10.16 ± 9.57 and 4.22 ± 5.47 after 3 rounds of onabotulinumtoxinA 200 units (*P* < 0.001 for both comparisons). In 22 patients, the onabotulinumtoxinA dose was increased to 200 units after just 3 rounds of 150 units therapy. These patients also tended to report less headaches after increase in the onabotulinumtoxinA dose, even though the *P* values did not reach significant levels.

**Table 2:** Trend of the headache days after increase in the onabotulinumtoxinA dose. (*n* = 175)

	Number of the headache days	Number of the severe headache days
Baseline	25.85 ± 5.73	15.20 ± 7.96
36 weeks before the increase	14.22 ± 10.81	5.88 ± 6.80
24 weeks before the increase	13.58 ± 10.91	5.46 ± 6.75
12 weeks before the increase	13.71 ± 10.62	5.32 ± 6.49
Day of the increase to 200 units	13.62 ± 10.79	5.88 ± 6.73
12 weeks after the increase	11.88 ± 10.55 *	4.33 ± 5.23 **
24 weeks after the increase	12.38 ± 10.69	3.83 ± 4.96 **
36 weeks after the increase	11.02 ± 10.61 **	4.01 ± 4.89 **

Data presented as mean ± SD; * *P* < 0.01, ** *P* < 0.001 for the paired t-test compared to the day of increase to 200 units

**Table 3:** Trend of the headache days by the duration of onabotulinumtoxin150 units therapy

	Number of the headache days	Number of the severe headache days
Patients who just had 3 rounds of onabotulinumtoxinA 150 units before the increase to 200 units (*n* = 22)		
Baseline (36 weeks before the increase)	25.86 ± 5.45	13.73 ± 8.54
24 weeks before the increase	19.41 ± 9.49	8.59 ± 9.11
12 weeks before the increase	17.40 ± 11.13	7.27 ± 9.15
Day of increase to 200 Units	15.95 ± 11.02	6.59 ± 6.90
12 weeks after the increase	15.09 ± 12.09	4.23 ± 5.77
24 weeks after the increase	13.59 ± 10.89	4.27 ± 3.99
36 weeks after the increase	14.77 ± 11.53	4.86 ± 4.11
Patients who had less than 8 rounds of onabotulinumtoxinA 150 units before the increase to 200 units (*n* = 92)		
Baseline	24.84 ± 6.13	14.26 ± 8.38
36 weeks before the increase	17.54 ± 10.90	6.92 ± 7.07
24 weeks before the increase	15.58 ± 11.53	5.74 ± 7.13
12 weeks before the increase	14.89 ± 11.22	5.08 ± 6.42
Day of increase to 200 units	14.36 ± 11.64	5.17 ± 5.92
12 weeks after the increase	12.86 ± 11.36	4.10 ± 4.90
24 weeks after the increase	12.81 ± 11.36	3.47 ± 4.11 **
36 weeks after the increase	11.79 ± 11.47 *	3.83 ± 4.32 **
Patients who had at least 8 rounds of onabotulinumtoxinA 150 units before the increase to 200 units (*n* = 83)		
Baseline	26.97 ± 5.04	16.25 ± 7.36
36 weeks before the increase	10.53 ± 9.48	4.72 ± 6.34
24 weeks before the increase	11.37 ± 9.77	5.16 ± 6.33
12 weeks before the increase	12.40 ± 9.93	5.59 ± 6.59
Day of increase to 200 units	12.79 ± 9.77	6.67 ± 7.48
12 weeks after the increase	10.79 ± 9.52 *	4.59 ± 5.59 **
24 weeks after the increase	11.90 ± 9.94	4.23 ± 5.76 ***
36 weeks after the increase	10.16 ± 9.57 **	4.22 ± 5.47 **

Data presented as mean ± SD; * *P* < 0.05, ** *P* < 0.01, *** *P* < 0.001 for the paired t-test compared to the day of increase to 200 units

Out of the 175 patients, 117 and 51 patients were followed for at least 60 (i.e., 5 rounds) and 84 weeks (i.e., 7 rounds) after the increase in the onabotulinumtoxinA dose, respectively (Table 4). On average these patients had 13.29 to 14.59 headache days per month while they were on 150 units of onabotulinumtoxinA. The number of the headache days reached as low as 10.51 ± 9.74 (*P* < 0.001) and 10.21 ± 9.19 (*P* < 0.01) days per month after the fifth and the seventh rounds of onabotulinumtoxinA 200 therapy, respectively. A similar trend existed for severe headache days. For at least up to 84 weeks after the increase in the onabotulinumtoxinA dose, patients continued to experience benefit from the dose increase (Table 4).

**Table 4:** Trend of the headache days in patients who were followed for over 36 weeks after the increase in the onabotulinumtoxinA dose

	Number of the headache days	Number of the severe headache days
Patients who were followed for at least 60 weeks after the increase in onabotulinumtoxinA dose (*n* = 117)		
Baseline	26.23 ± 5.63	15.31 ± 7.83
36 weeks before the increase	13.89 ± 10.69	5.37 ± 6.01
24 weeks before the increase	13.35 ± 10.41	5.34 ± 6.40
12 weeks before the increase	13.56 ± 10.39	4.99 ± 6.27
Day of increase to 200 units	13.40 ± 10.37	5.28 ± 6.03
12 weeks after the increase	12.07 ± 10.40	4.47 ± 5.69
24 weeks after the increase	13.18 ± 10.77	3.80 ± 4.98 **
36 weeks after the increase	11.14 ± 10.48 **	3.67 ± 4.82 ***
48 weeks after the increase	12.12 ± 10.70	4.02 ± 5.51 *
60 weeks after the increase	10.51 ± 9.74 ***	3.55 ± 4.93 ***
Patients who were followed for at least 84 weeks after the increase in onabotulinumtoxinA dose (*n* = 51)		
Baseline	27.21 ± 4.86	17.08 ± 7.87
36 weeks before the increase	13.29 ± 10.78	5.08 ± 5.47
24 weeks before the increase	14.33 ± 10.55	6.18 ± 6.89
12 weeks before the increase	13.61 ± 10.70	5.37 ± 6.08
Day of increase to 200 units	14.59 ± 10.63	5.00 ± 5.84
12 weeks after the increase	13.67 ± 10.81	4.78 ± 5.75
24 weeks after the increase	14.84 ± 10.73	3.63 ± 4.27
36 weeks after the increase	11.41 ± 10.51 *	3.61 ± 4.39 *
48 weeks after the increase	13.49 ± 11.13	3.57 ± 3.53
60 weeks after the increase	10.12 ± 9.70 **	3.92 ± 5.09
72 weeks after the increase	11.72 ± 10.84	3.84 ± 4.69
84 weeks after the increase	10.21 ± 9.19 **	3.16 ± 4.04 *

Data presented as mean ± SD; * *P* < 0.05, ** *P* < 0.01, *** *P* < 0.001 for the paired t-test compared to the day of increase to 200 units

Table 5 reports headache days stratified by the location of the most intense pain. Overall, patients who did not report a specific location for their headaches and had holocephalic pain tended to have more intense headaches compared to the people who were able to identify a location with most intense pain. The favorable effect of the 200 units compared to the 150 units of the onabotulinumtoxinA remained statistically significant in the frontal and temporoparietal headache groups (*P* < 0.05). In patients with occipitonuchal and holocephalic headaches, onabotulinumtoxinA 200 units tended to be superior to 150 units, even though the difference did not reach significant levels. When these two groups were combined and analyzed together, the difference again became statistically significant (*P* < 0.05).

**Table 5:** Trend of the headache days by the location of the most intense pain

	Number of the headache days	Number of the severe headache days
Frontal (*n* = 73)		
Baseline	26.11 ± 5.23	15.42 ± 8.15
36 weeks before the increase	14.51 ± 11.09	5.90 ± 6.74
24 weeks before the increase	14.01 ± 11.27	5.11 ± 6.15
12 weeks before the increase	13.08 ± 11.47	5.83 ± 7.44
Day of increase to 200 units	13.19 ± 11.33	5.84 ± 7.31
12 weeks after the increase	12.05 ± 11.15	4.68 ± 6.33 *
24 weeks after the increase	13.41 ± 11.11	4.57 ± 6.44
36 weeks after the increase	11.37 ± 11.25 *	4.08 ± 5.77 **
Temporoparietal (*n* = 51)		
Baseline	26.16 ± 5.63	15.37 ± 7.80
36 weeks before the increase	14.06 ± 10.85	5.25 ± 5.69
24 weeks before the increase	12.35 ± 10.28	5.20 ± 5.44
12 weeks before the increase	12.45 ± 9.62	5.08 ± 5.04
Day of increase to 200 units	14.16 ± 10.43	6.00 ± 5.16
12 weeks after the increase	11.86 ± 9.78 *	4.18 ± 4.59 *
24 weeks after the increase	11.02 ± 10.24 **	2.98 ± 3.66 ***
36 weeks after the increase	11.14 ± 9.77 *	4.02 ± 4.10 *
Occipitonuchal (*n* = 28)		
Baseline	23.50 ± 6.75	13.39 ± 6.62
36 weeks before the increase	10.75 ± 9.59	3.93 ± 4.33
24 weeks before the increase	12.86 ± 11.13	5.36 ± 7.36
12 weeks before the increase	12.53 ± 10.24	3.82 ± 5.77
Day of increase to 200 units	11.57 ± 9.55	4.36 ± 6.59
12 weeks after the increase	8.61 ± 9.15	3.07 ± 3.94
24 weeks after the increase	9.78 ± 9.65	2.61 ± 2.38
36 weeks after the increase	7.39 ± 9.01	2.79 ± 3.33
Holocephalic (*n* = 23)		
Baseline	27.22 ± 5.71	16.35 ± 9.26
36 weeks before the increase	17.87 ± 10.54	9.56 ± 10.04
24 weeks before the increase	15.83 ± 11.10	7.30 ± 8.45
12 weeks before the increase	16.74 ± 10.73	6.04 ± 6.95
Day of increase to 200 units	16.26 ± 11.30	7.65 ± 7.92
12 weeks after the increase	15.35 ± 11.26	5.09 ± 3.89
24 weeks after the increase	15.30 ± 11.03	4.83 ± 3.93
36 weeks after the increase	14.04 ± 11.53	5.26 ± 4.97
Occipitonuchal and holocephalic (*n* = 51)		
Baseline	25.18 ± 6.51	14.72 ± 7.98
36 weeks before the increase	13.96 ± 10.55	6.47 ± 7.91
24 weeks before the increase	14.20 ± 11.10	6.23 ± 7.85
12 weeks before the increase	14.43 ± 10.57	4.82 ± 6.36
Day of increase to 200 units	13.69 ± 10.54	5.84 ± 7.34
12 weeks after the increase	11.65 ± 10.61	3.98 ± 4.01
24 weeks after the increase	12.27 ± 10.56	3.61 ± 3.33 *
36 weeks after the increase	10.39 ± 10.66 *	3.90 ± 4.29 *

Data presented as mean ± SD; * *P* < 0.05, ** *P* < 0.01, *** *P* < 0.001 for the paired t-test compared to the day of increase to 200 units

Total of 146 patients were on the same number of preventative medications 36 weeks before and at the time of escalation of the onabotulinumtoxinA dose. As shown in Table 6, onabotulinumtoxinA 200 units remained to be superior to 150 units irrespective to the change in the number of the headache preventative medications (*P* < 0.05).

**Table 6:** Trend of the headache days by change in the number of the headache preventative medications

	Number of the headache days	Number of the severe headache days
Patients who were on the same number of the preventative medications (*n* = 146)		
Baseline	25.94 ± 5.77	15.54 ± 8.04
36 weeks before the increase	13.55 ± 11.04	5.66 ± 6.97
24 weeks before the increase	12.67 ± 10.78	5.19 ± 6.86
12 weeks before the increase	13.04 ± 10.73	5.00 ± 6.46
Day of increase to 200 units	12.81 ± 10.66	5.42 ± 6.26
12 weeks after the increase	10.85 ± 10.36 **	3.77 ± 4.71 ***
24 weeks after the increase	11.70 ± 10.45	3.59 ± 4.73 ***
36 weeks after the increase	10.36 ± 10.46 **	3.70 ± 4.73 ***
Patients who were on different number of the preventative medications (*n* = 29)		
Baseline	25.41 ± 5.56	13.52 ± 7.44
36 weeks before the increase	17.55 ± 9.02	7.00 ± 5.85
24 weeks before the increase	18.17 ± 10.51	6.83 ± 6.08
12 weeks before the increase	17.07 ± 9.89	6.90 ± 6.49
Day of increase to 200 units	17.69 ± 10.69	8.21 ± 8.47
12 weeks after the increase	17.07 ± 10.12	7.17 ± 6.74
24 weeks after the increase	15.79 ± 11.42	5.00 ± 5.96 *
36 weeks after the increase	14.34 ± 10.92 *	5.55 ± 5.43 *

Data presented as mean ± SD; * *P* < 0.05, ** *P* < 0.01, *** *P* < 0.001 for the paired t-test compared to the day of increase to 200 units

Table 7 reports the wearing off period after different doses of onabotulinumtoxinA. After excluding new patients and those who had > 25 headache days at the time of increase and/or 36 weeks after the increase of the onabotulinumtoxinA, patients reported less wearing off days while they were on 200 units of onabotulinumtoxinA (14.68 ± 9.21 vs. 16.91 ± 10.36 days, *P* < 0.05, Table 7).

**Table 7:** Trend of the wearing off periods after increase in the onabotulinumtoxinA dose. (*n* = 113)

	Wearing off period, days
36 weeks before the increase	16.11 ± 9.11
24 weeks before the increase	15.67 ± 11.08
12 weeks before the increase	16.54 ± 11.94
Day of increase to 200 Units	16.91 ± 10.36
12 weeks after the increase	14.00 ± 10.75 *
24 weeks after the increase	13.26 ± 9.13 **
36 weeks after the increase	14.68 ± 9.21 *

Data presented as mean ± SD; * *P* < 0.05, ** *P* < 0.01 for the paired t-test compared to the day of increase to 200 units

## Discussion

The data presented in this paired comparison study favor the onabotulinumtoxinA 200 units over 150 units in headache control. The beneficial effect of the higher dose of onabotulinumtoxinA persisted after patients were stratified by different confounding variables like duration of the onabotulinumtoxinA therapy, headache location and change in the number of the headache preventative medications. The favorable effect of the higher dose of onabotulinumtoxinA extended to the wearing off period as well.

In the PREEMPT 1, onabotulinumtoxinA therapy led to a significant improvement in the headache days (− 7.8 and − 6.4 days after onabotulinumtoxinA and placebo treatments, respectively) and migraine days (− 7.6 and − 6.1 days after onabotulinumtoxinA and placebo treatments, respectively) [20]. Similarly, PREEMPT 2, demonstrated significant decrease in the headache days (− 9.0 and − 6.7 days after onabotulinumtoxinA and placebo treatments, respectively) and migraine days (− 8.7 and − 6.3 days after onabotulinumtoxinA and placebo treatments, respectively) [21]. Although both trials favored onabotulinumtoxinA therapy at the dose of 155–195 units for headache control, individuals in placebo arms also experienced significant improvement in the headache related variables [20, 21]. In a small study, Cady et al. reported 10.1 and 5.2 day decrease in the number of migraine days in the onabotulinumtoxinA and placebo groups, respectively [22]. In another study, onabotulinumtoxinA-treated patients had 2 less headache days than those who received placebo treatment [23]. A systematic review of onabotulinumtoxinA therapy in migraine patients concluded that onabotulinumtoxinA reduces the number of the migraine days by 2 days per month; this conclusion was based on the moderate quality of the evidence from the two PREEMPT trials [24]. In the same systematic review, the rest of the clinical trials were rated to have very low to low quality of the evidence [24]. Interestingly, our results showed that increase in the onabotulinumtoxinA dose improves the number of headache days by around 2 days. This value is similar to the amount of the improvement after onabotulinumtoxinA vs. placebo treatments in the abovementioned trials. The improvement after higher dose of onabotulinumtoxinA persisted and even gradually progressed as patients continued to receive the higher dose. Sensitivity analysis showed that the subjects, who have been on onabotulinumtoxinA 150 units for over 2 years, also experience a significant drop in their headache days after increase in the onabotulinumtoxinA dose.

There are controversial data about the onabotulinumtoxinA effective dose in headache prevention. In PREEMPT trials, authors did not evaluate the efficacy of the different doses of onabotulinumtoxinA, instead they compared the onabotulinumtoxinA therapy at the doses ranging from 155 to 195 units to the placebo treatment [20, 21]. Silberstein et al., in a randomized clinical trial compared different doses of the onabotulinumtoxinA (i.e. 75, 150 and 225 units) to the placebo treatment [25]. After 240 days, patients who received 150 or 225 units of onabotulinumtoxinA experienced significant decrease in the headache frequency. This difference did not reach significant levels for the group treated with onabotulinumtoxinA 75 units. Nevertheless, no group was consistently superior in their study [25]. In another study, patients who did not respond to the conventional onabotulinumtoxinA therapy (mean dose ± SD: 170 ± 10 units) were enrolled to receive six rounds of IncobotulinumtoxinA 200 units followed by two rounds of 200 units of onabotulinumtoxinA [26]. In that study, higher dose of botulinumtoxinA therapy showed sustained benefit in the frequency and severity of the monthly headache episodes [26]. Similarly, 195 units of onabotulinumtoxinA was superior to 155 units in a small study on Russian women [27]. Our findings suggest the dose-dependent response to onabotulinumtoxinA therapy. Notably, in our patients after three rounds of onabotulinumtoxinA 200 units, the decrements in the total number of the headache days and severe headache days were similar and were around 2 days. This suggests that the higher onabotulinumtoxinA dose primarily reduces the severe headache days rather than the milder headaches which are less debilitating.

After dividing the patients by the headache location, the beneficial effect of the higher onabotulinumtoxinA persisted in patients with frontal and temporoparietal headaches. In patients with occipitonuchal and holocephalic headaches, although there was a trend favoring higher dose of onabotulinumtoxinA, the difference did not reach significant levels; perhaps because of the small sample sizes and limited statistical power. When these two groups were combined and analyzed together, again the difference between 200 and 150 units of onabotulinumtoxinA became statistically significant. Based on the PREEMPT injection protocol, it has been recommended that clinicians use the “follow-the-pain” strategy and inject up to 40 additional units of onabotulinumtoxinA in 8 different injection sites [11]. Our study suggests that the beneficial effect of the higher dose of onabotulinumtoxinA is independent from the headache location and the sites of the injection. For instance, patients with frontal headaches reported less headaches on 200 units of OnabotulinumtoxinA, even though the extra 50 units were injected into their temporalis and occipitalis muscles and they continued to receive the same dose of onabotulinumtoxinA on their forehead. Our results dispute the “follow-the-pain” strategy. We suggest clinicians do not necessarily need to use the “follow-the-pain strategy” in order to improve the headache control. This is especially important when injecting extra units of onabotulinumtoxinA to a certain location like forehead, can increase the risk of adverse events such as ptosis.

The antinociceptive duration of onabotulinumtoxinA therapy is not well known. It is supposed that the duration is similar to the neuromuscular blockade effect of onabotulinumtoxinA (i.e. 12 weeks), even though its analgesic effect is unrelated to the inhibition of the neuromuscular transmission [28, 29]. About half of the patients with chronic migraine, report sooner than expected wearing off of the antinociceptive effects of the onabotulinumtoxinA therapy [30]. In this subgroup of patients, the wearing off period is estimated to be around 18 days, and is associated with increased consumption of the analgesic medications [30]. Although individual-based adjustment of the treatment interval is suggested for certain neurological conditions, shorter onabotulinumtoxinA treatment interval increases the risk of neutralizing antibody formation and may result in the treatment failure [31, 32]. Our results show that the wearing off period is inversely associated with the dose of the onabotulinumtoxinA. Because patients with poor headache control may not be able to reliably report the wearing off period, we excluded those who had over 25 headache days at certain times during the course of the study. Given that patients with long wearing off periods are generally recommended against having onabotulinumtoxinA sooner than the regular 12 week interval schedule, increase in the onabotulinumtoxinA may be considered as an alternative approach to improve the headache control in this subset of patients.

The financial burden of migraine is substantial on patients and their employers. The total annual direct plus indirect healthcare costs are around $9000 higher in migraine patients compared to their non-migraine counterparts [33]. Chronic migraine is also associated with higher healthcare cost than episodic migraine [34]. The migraine indirect costs are mostly related to the loss of productivity during the patients’ peak employment years [33], and have larger economic impact than direct costs especially in patients with severe migraine [35]. In chronic migraine, indirect costs constitute around 70% of the total migraine related costs [36]. Hu et al. estimated $13.3 billion migraine related indirect costs and 3.8 and 8.3 missed workdays and 7.5 and 7.6 impaired working days annually in American men and women, respectively [37]. In a Canadian population based study, 77% of migraineurs reported restriction in their functional ability, 50% had to discontinue the normal activities, 30% had to cancel family or social activities, and 19% reported missing work time during their last migraine attack [38]. OnabotulinumtoxinA is a cost-effective treatment for chronic migraine and is associated with decreased healthcare utilization [39, 40]. Severe headache days have more impact on the function and productivity of patients and hence impose higher financial burden on the patients and employers. We found that increase in the dose of onabotulinumtoxinA, reduces the number of severe headache days by around 2 days or 30%. This improvement can lead to less missed and impaired working days, which by itself improves the productivity of patients and migraine costs.

This study has several limitations, which should be considered when interpreting the results. The main limitation is that the patients were not blinded to the dose of the onabotulinumtoxinA therapy. Before the increase in the dose, patients already had at least 3 rounds of onabotulinumtoxinA 150 units and were not satisfied with their headache control. Overall, blinding an onabotulinumtoxinA study is very challenging. In the Australian public assessment report for onabotulinumtoxinA, over 80% of the individuals in the onabotulinumtoxinA arm, were able to correctly guess that they were getting the active medication and not the placebo (https://www.tga.gov.au/sites/default/files/auspar-botox.pdf). This can lead to placebo effect. We controlled this potential source of bias, by following a subset of our patients for 84 weeks after the increase in the onabotulinumtoxinA dose. Unlike the response usually seen due to placebo effects, which would decrease with continuation of therapy; our patients improved further as they continued to receive the higher dose of the onabotulinumtoxinA. The other limitation is that, there was insufficient information in the questionnaires to determine the number of the migraine days; instead we recorded the number of the severe headache days, which was self-defined. Because of the abovementioned limiting factors, future randomized clinical trials are needed to confirm our findings.

## Conclusions

This is the first paired comparison study, which evaluates the response to different doses of the onabotulinumtoxinA therapy. We found that higher dose of onabotulinumtoxinA leads to sustained improvement in headache control. This effect is independent from duration of the therapy, and headache location. Hence, patients with unsatisfactory response to the conventional 150 units dose of onabotulinumtoxinA may gain benefit from higher doses. Although this study suggests doses-dependent response to onabotulinumtoxinA, our findings need to be confirmed by future randomized double-blinded clinical trials.

## Data Availability

The anonymized patient level data are available upon request from the corresponding author, Fred Michael Cutrer.

## Abbreviations

BMI: Body mass index
PREEMPT: Phase 3 REsearch Evaluating Migraine Prophylaxis Therapy
SD: Standard deviation
